# Neuroregenerative effects of olfactory ensheathing cells transplanted in a multi-layered conductive nanofibrous conduit in peripheral nerve repair in rats

**DOI:** 10.1186/s12929-015-0144-0

**Published:** 2015-05-20

**Authors:** Mahboubeh Kabiri, Saeed Oraee-Yazdani, Abbas Shafiee, Hana Hanaee-Ahvaz, Masumeh Dodel, Mohammad Vaseei, Masoud Soleimani

**Affiliations:** Department of Biotechnology, College of science, University of Tehran, Tehran, Iran; Department of Stem Cell Biology, Stem Cell Technology Research Center, Tehran, Iran; Department of Nanotechnology and Tissue Engineering, Stem Cell Technology Research Center, Tehran, Iran; Functional Neurosurgery Research Center, Department of Neurosurgery, Shohada Tajrish Hospital, Shahid Beheshti University of Medical Sciences, Tehran, Iran; Experimental Dermatology Group, UQ Centre for Clinical Research, The University of Queensland, Herston, Queensland Australia; Department of Textile engineering, Amirkabir University of Technology, Tehran, Iran, Stem Cell Technology Research Center, Tehran, Iran; Pathology Department, Shariati Hospital, Tehran University of Medical Sciences, Tehran, Iran; Department of Hematology, Faculty of Medical Science, Tarbiat Modares University, Tehran, Iran

**Keywords:** Nerve tissue engineering, Olfactory Ensheathing Cells, Composite scaffold, Conductive, Nanofibrous conduit, Carbon nanotube

## Abstract

**Background:**

The purpose of this study was to evaluate the efficacy of a multi-layered conductive nanofibrous hollow conduit in combination with olfactory ensheathing cells (OEC) to promote peripheral nerve regeneration. We aimed to harness both the topographical and electrical cues of the aligned conductive nanofibrous single-walled carbon nanotube/ poly (_L_-lactic acid) (SWCNT/PLLA) scaffolds along with the neurotrophic features of OEC in a nerve tissue engineered approach.

**Results:**

We demonstrated that SWCNT/PLLA composite scaffolds support the adhesion, growth, survival and proliferation of OEC. Using microsurgical techniques, the tissue engineered nerve conduits were interposed into an 8 mm gap in sciatic nerve defects in rats. Functional recovery was evaluated using sciatic functional index (SFI) fortnightly after the surgery. Histological analyses including immunohistochemistry for S100 and NF markers along with toluidine blue staining (nerve thickness) and TEM imaging (myelin sheath thickness) of the sections from middle and distal parts of nerve grafts showed an increased regeneration in cell/scaffold group compared with cell-free scaffold and silicone groups. Neural regeneration in cell/scaffold group was very closely similar to autograft group, as deduced from SFI scores and histological assessments.

**Conclusions:**

Our results indicated that the tissue engineered construct made of rolled sheet of SWCNT/PLLA nanofibrous scaffolds and OEC could promote axonal outgrowth and peripheral nerve regeneration suggesting them as a promising alternative in nerve tissue engineering.

## Backgrounds

Peripheral nerve injuries (PNI) occur in approximately 3 % of all trauma patients [1, 2] typically causing a debilitating disease with lifelong suffering and disturbances in functions. In small segmental injuries with no gap, the nerve defect can be repaired by surgical coaptation of the two nerve bundles, provided no excess tension is placed on the nerve ends patients [1]. In the case of a gap between the two ends, nerve grafting is required to bridge the void space [3]. In large defects, the autologous nerve graft is considered as the gold standard for peripheral nerve repair. However, this strategy suffers from limited availability of donor nerves and second-site morbidity [3]. A fundamental approach for nerve repair is neural tissue engineering which involves fabricating polymeric scaffolds with suitable neural or glial cells with or without growth factors to produce a perfectly suited three-dimensional nerve graft for implantation to the lesion site. In this regard, scaffold design is of pivotal importance as it creates the environment for controlling cell behavior, cell attachment and proliferation that also guides the direction of newly sprouted axons and migration of cells towards or outwards the graft [4]. A tremendous body of research has shown the suitability of natural and synthetic polymers to support neural tissue development and their capacity to guide the regrowth of nerve tissue [5]. These materials have been used in the form of single hollow lumen or more complex structures such as multi-channel nerve guidance conduits (NGC) or NGC with lumen fillers [6]. Non-degradable silicon tubes, acting as a simple hollow lumen, were the first biomaterials clinically used to bridge the nerve gaps. However, they often require second surgery for removal and may become detriment due to infection or foreign body reaction [7]. Of biodegradable materials, poly (_L_-lactide coglycolide) (PLGA), polyglycolic acid, poly (3-hydroxybutyrate), collagen, chitosan and silk have been thoroughly investigated as a substrate for NGC [5]. Although a wide range of successes have been achieved in this field, the amount of nerve repair is still unsatisfactory due to their inappropriate physical properties or lack of biological cues [8]. As such, fabrication of composite scaffolds with aiming to further tune the architecture and topographical and physicochemical properties of the neural scaffold along with endowing the scaffold with biological cues viaincorporation of growth factors or transplantation of growth promoting cells has been tried out to further improve the nerve tissue engineering approach [5].

In recent years, olfactory ensheathing neuroglial cells (OEC) have gradually shown their therapeutic potential for the treatment of spinal cord injuries (SCI). Basically their main role is to facilitate and guide the regeneration of non-myelinated olfactory axons from the peripheral nasal epithelium to the hostile olfactory bulb in the forebrain [9]. OEC have a dual nature of astroglial cells (regarding anatomical location and glial fibrillary acidic protein expression) and Schwann cells (SC) (regarding axonal ensheathment and myelination and neurotrophics expression) [10, 11]. Transplantation of each of these cell types (OEC and SC) to the SCI site has yielded satisfactory results and has opened windows of hope for patients suffering from SCI [12]. After transplantation, Schwann cells can induce functional recovery, axon regeneration and myelination and reduce cyst formation and secondary damages to the tissue [13]. Similarly OEC have shown remarkable capabilities to reduce scar and cavity formation and promote regeneration after SCI [14–16]. Despite of being a constituent of both peripheral and central nervous sysytem, OEC have mostly been investigated as supportive cells for SCI and very few reports have addressed their usefulness for PNI. OEC can secrete neurotrophic factors, remyelinate damaged axons, survive and migrate within the nerve tissue and guide the axonal neurite outgrowth [17]. Regarding the availability of patient specific OEC, their neurotrophic characteristics and their safety and effectiveness for the treatment of nerve injuries [15] these cells may be great candidates for implantation to PNI to promote neurite extension, endogenous Schwann cell migration and finally better nerve regeneration [18]. In comparison with SCI, OEC have been underappreciated for PNI and just a few studies have addressed their incredible therapeutic potential [19, 18]. Yet, much less studies have utilized these cells in a tissue engineering paradigm [20].

Poly (α-hydroxy esters), such as poly (_L_-lactic acid) (PLLA) and its breakdown products are shown to be biocompatible with neural cells and tissues both *in vitro* and *in vivo* [5]. Their tunable degradation rate, the non-immunogenicity and FDA approval has made them enormously attractive in tissue engineering approaches [21]. Electrospinning these polymers allows for the generation of aligned fibers with diameters in the nano-meter range that are suitable in directed axonal outgrowth through provision of appropriate contact guidance [22, 21]. The direction of nerve cell elongation and axon outgrowth is dictated by the direction of fibers of the substratum [23]. The aligned nanofibrous scaffold can present the newly formed axons with an intricate topography with a positive cue to direct neurite outgrowth to the distal part of the injured nerve. Furthermore, the electrospun nanofibrous sheets have the capacity to be rolled and packed within a defined volume, providing enough substrate for cell transplantation. We hypothesized that further functionalizing of the PLLA nanofibers with an electrically conductive compound can aid to mimic the inherently conductive nature of the nerve tissues. Electrically conductive materials such as polypyrrole, polyaniline and carbon nanotubes (CNT) have been effectively used in drug delivery and biosensor applications and for the fabrication of NGC in nerve tissue engineering [24–28]. The resultant conductive composite would inherit the physical properties of polymeric materials and the electrical characteristics of the conductive material needed for specific applications such as nerve tissue engineering. Electrical stimulation has previously been shown to guide axon orientation and direct neurite extension [25], outlining the importance of the conductive substrate in enhanced nerve regeneration applications.

In the present study we aimed to harness both the topographical and electrical cues of the aligned nanofibrous CNT incorporated PLLA composite scaffolds, designed as both a guidance conduit and a cell delivery platform, and also the desirable neurotrophic features of OEC in the regeneration of transected sciatic nerves in rats. For this purpose we fabricated conductive nanofibrous composite scaffolds of SWCNT and PLLA, and seeded them with OEC to exploit their promising regenerative potentials. Following characterization of the scaffold, we evaluated its biocompatibility and *in vivo* peripheral nerve regeneration capacity of the cell-scaffold construct.

## Methods

### Scaffold fabrication and characterization

Electrospinning was used to fabricate composite scaffolds of SWCNT and PLLA. PLLA (M_W_ = 157000, Sigma-Aldrich) was dissolved in a solvent mixture of chloroform and N, N-dimethylformamide (DMF) (8.5:1.5, v/v) to have a final concentration of 3.5 % w/v. SWCNT (Plasmachem) nanoparticles were first well dispersed in chloroform to form a homogenous suspension, and then combined with DMF and PLLA in the proportions stated above. The final concentration of SWCNT in solution was equivalent to 3 % of the PLLA mass. The polymer solution was ultrasonicated and stir homogenized overnight before electrospinning. A syringe pump was used to feed the solution through an extension tube ended in a blunted 21-gauge needle. A voltage potential of 25 kV was applied between the needle and the collector. The nanofiber jet was collected on a stainless steel cylinder rotating at 2400 RPM at a fixed distance of 15 cm from the spinneret tip. Oxygen plasma surface treatment was performed using a low frequency plasma generator set on 40 kHz (Diener Electronics). The hydrophobic/hydrophilic nature of the nanofiber scaffolds before and after plasma treatment was evaluated by measuring the contact angle of water droplets using the sessile drop method (G10 contact angle goniometer, Kruss).

The morphology of the nanofibrous scaffolds and the surface characteristics of cell seeded scaffolds were evaluated by scanning electron microscopy (SEM) (Philips Xl-30). The scaffold or cell/scaffold constructs were washed thoroughly with phosphate buffered saline (PBS) and fixed with 2.5 % glutaraldehyde solution for two hours. The dehydrated samples were sputter coated with gold for 90 s and deposited onto SEM stubs.

### OEC isolation and culture

OEC were obtained from olfactory bulbs of adult *gfp* rats. The olfactory bulb was dissected, finely minced and digested in collagenase/dispase II solution as previously described in details [29]. After enzyme deactivation the tissue was mechanically triturated and cells were spun down with centrifugation. The cell pellet was cultured in DMEM/F12 (Gibco) supplemented with 10 % fetal bovine serum (FBS, Sigma), 2 mM L-glutamine (Gibco), 100 IU/ml penicillin and 100 μg/ml streptomycin (Gibco). Cultures were incubated in 5 % CO_2_ incubator for 18 hours. Next the supernatant was cultured for another 36 hours in a new uncoated flask. The supernatant was then used to feed poly-L-lysine (Sigma) coated flasks, knowing most of the OEC have not attached to the previous uncoated flasks. Cells were cultured on poly-l-lysine coated plates until appropriate cell number was achieved.

### Cell seeding on the scaffold

Scaffolds were sterilized in 70 % ethanol for 2 hours and then rinsed copiously with PBS prior to use in culture. The biocompatibility of composite scaffolds was assessed by examining cell proliferation. To investigate cell morphology of the OEC on aligned nanofibers, cell-scaffold constructs were fixed with 2.5 % glutaraldehyde solution. The specimens were then undergone dehydration using series of graded ethanol solutions. The dried samples were then sputter coated with gold and observed with SEM.

For in vivo studies, OEC were preseeded on electrospun nanofibrous sheets and grown until confluence. The sheets were rolled to make a cylindrical conduit with 4 to 5 layers of cell-seeded walls. Approximately 20000 cells were transplanted into each rat in cell/scaffold group. We used this scaffolding system to bridge the gap between the remaining functional tissues. The two nerve stumps were placed and encapsulated within the CNT/PLLA composite conduit or silicon tube, or were sutured end to end in the autograft gold standard group. After 9 weeks the animals were killed and the sciatic nerve was removed for histological studies.

### Animals

Adult Sprague–Dawley rats (250–300 g) were obtained from the Razi Vaccine and Serum Research Institute, Iran. All experimental procedures were approved by the Animal Care and Ethics of Stem Cell Technology research centre, Iran. Stem Cell Technology research center guidelines for the care and use of laboratory animals have been observed. Rats were housed with 12-h light and dark cycles. Food and water was available ad libitum in the cages. Animals were randomly divided into four groups (seven rats each): I: a group in which an eight mm of left sciatic was dissected out and sutured in opposite direction (autograft), II: a group receiving cell seeded nanofibrous conduit (cell/scaffold), III: a group receiving non-seeded conduit (scaffold) and IV: a control group receiving 1 mm inner diameter silicone tube (silicone).

### Surgeries

The rats were anesthetized with an intra-peritoneal injection of a solution containing ketamine (50 mg/kg) plus xylazine (5 mg/kg). The surgical area was shaved and sterilized with betadine. Rats were kept on a heating pad at 37 °C during surgery. After opening the skin and muscle layers, left sciatic nerve was exposed and an eight mm segment was resected. A 10 mm long graft was interposed in between the two severed ends of the sciatic nerve. In order to reestablish the continuity of the nerve, in autograft group the nerve and the graft were sutured end to end. In the rest of the groups, both the proximal and distal ends of the sciatic nerve were inserted inside the conduit (CNT/PLLA or silicone conduits, 1 mm in), and fixed with two 10-0 nylon epineural sutures. Then the muscle and skin were sutured and the animals recovered in a controlled environment. All rats received cefazolin (40 mg/kg) and gentamicin (12 mg/kg).

### Walking track analysis

Animals were evaluated fortnightly to obtain sciatic functional index (SFI) values which recall the performance of the hind limb movement. Different footprint parameters were measured and incorporated in the formula developed by Bain et al [30]. These parameters include PL: the print length (distance from the heel to the third toe); TS: the toe spread (distance between the first and the fifth toe); ITS: the intermediary toe spread (the distance between the second and the fourth toe). These parameters were collected from both the experimental feet (E) and the normal (N) feet serving as internal control. The SFI values were obtained according the following formula:

$$ \mathrm{S}\mathrm{F}\mathrm{I} = -38.{3}^{\mathrm{x}}\mathrm{P}\mathrm{L}\mathrm{F} + 109.{5}^{\mathrm{x}}\mathrm{T}\mathrm{S}\mathrm{F} + 13.{3}^{\mathrm{x}}\mathrm{I}\mathrm{TS}\mathrm{F} - 8:8 $$

In which the PLF is the print length factor calculated as (EPL-NPL)/NPL; TSF is the toe spread factor, calculated as (ETS-NTS)/NTS and the ITS is the intermediary toe spread factor measured as (EITS-NITS)/NITS. The calculated SFI from the above formula ranges between a zero score, reflecting normal function, to -100 theoretically representing total impairment. The footprints were obtained by dipping rat’s hind paws on an ink blotter and placing them on a walking pathway. The bottom of the track was covered with a white piece of paper. Each foot produced four to five prints.

### Histological analysis of nerve grafts

Nine weeks post-transplantation, all the rats were administered an ip overdose of ketamine (70 mg/kg) and xylazine (10 mg/kg). The implant (autograft or nerve conduits) was then harvested and fixed overnight in 4 % paraformaldehyde (0.1 M, pH 7.4) for histological staining. The distal parts of the nerve explants were post-fixed in 1 % osmium tetroxide. Semi-thin sections were obtained from resin embedded samples and stained with 0.1 % toluidine blue (TB). Some ultra-thin sections (100 nm) of the mid parts of the nerve conduits were prepared for ultrastructure evaluation with transmission electron microscope (Philips EM208S). The middle parts were paraffin embedded and 5 micrometer thick cross sections of the sciatic nerve were cut and mounted on poly-l-lysine coated slides. Sections were processed for immunostaining with monoclonal anti-neurofilament (NF, Sigma), anti-S100 (Chemicon) or anti-*gfp* (Abcam) antibody, followed by incubation with HorseRadish Peroxidase conjugated secondary antibody (Abcam). Samples were then examined under light microscope after adding diaminobenzidine (Sigma).

### Image analysis and statistical measurements

To quantify nerve regeneration, the nerve fiber diameter, the axonal diameter and the myelin sheath thickness were measured using Image Pro Plus 6.0 software (Media Cybernetics). Statistical comparison between the groups was conducted using Oneway ANOVA followed by Dunnett T3 as post hoc test. The comparative analyses were done using SPSS version 13.

## Results

### Cell seeding on the scaffold

**Fig. 1:**
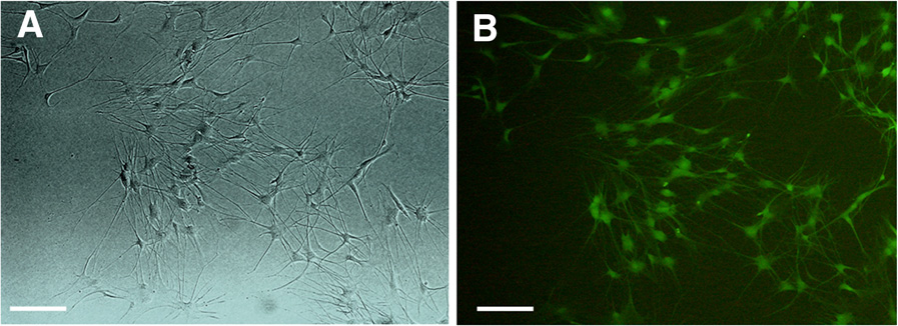
Morphology of isolated OEC. (**a**) *in vitro* morphology of isolated cells using light microscopy; (**b**) *gfp* expressing OEC, The cells show a bipolar or three polar characteristic mostly connected with each other; Scale bars 10 μm

**Fig. 2:**
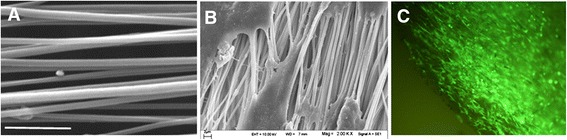
Effect of guidance cues on the alignement of OEC. (**a**) Aligned SWCNT/PLLA nanofibers used as the substratum for OEC, scale bar = 10 μm; (**b**) SEM micrographs of OEC aligned on nanofiber SWCNT/PLLA scaffolds, scale bar = 2 μm; (**c**) Fluorescence image of aligned OEC grown on SWCNT/PLLA nanofibrous scaffolds, magnification 100x

OEC were isolated from olfactory bulb of *gfp* Sprauge-Dawley rats. The isolated cells were first phenotypically characterized by flow cytometery and immunofluerescence assay indicating the purity of the cells (using CD271 neurothrophin receptor) to be about 80 % (unpublished observation). OEC populations are composed of adherent cells with heterogeneous morphologies, but mostly display spindle or polygonal shape (Fig. 1a, b). The nanostructure of SWCNT doped PLLA scaffold is shown in Fig. 2a. These nanofibers have a narrow range of diameter distribution with an average of ~430 nm.We observed an excellent increase in hydrophilicity of the nanofibers after modification with oxygen plasma, with a reduction in water contact angle from 137 to a non-detectable amount. In deed such surface modifications are commonly used to enhance protein adsorption and subsequent cell attachment onto the scaffolds. We have previously shown that addition of 3 % w/w CNT to the PLLA fibers can dramatically enhance its electrical conductive properties with minimal CNT agglomeration in the fiber constructs and no cytotoxic effects on cellular behavior [31]. The composite scaffold used in this study was shown to have nanometer diameter fibers, well aligned orientation and high conductivity combining appropriate properties for a substratum intended for nerve tissue engineering applications. The attachment of OEC onto the nanofibrous scaffolds provides a qualitative assessment of the biocompatibility of the composite fibers. According to Fig. 2b, c, as OEC proliferate, they get aligned along the direction of the fibers. As shown in SEM micrographs, OEC tightly attached and followed the orientation of the fibers. Our results indicate that the aligned nanofiber composites can provide contact guidance cues to direct cell alignment. This strategy can have promising implications in guided peripheral nerve regeneration.

### Walking track assessment

The parameters used in evaluation of functional restoration in sciatic nerve injuries, assessed by SFI, are foot print length, toe spread and intermediary toe spread. Such an index correlates with the level of reinnervation of the intrinsic muscle of the feet. The SFI values are listed in Table 1. In all groups the SFI dropped from 0 to about -100 after sciatic nerve transection, showing complete loss of motor function, and then progressively improved with time but with differing degrees. In none of the treatments the walking pattern showed a complete recovery, but the SFI improved in a more marked way in autograph and cell/scaffold groups. In the two remaining groups the SFI improved slightly, but there was no significant difference between them throughout the follow-up period. Cell/scaffold group showed a significantly better recovery compared to scaffold and silicone groups at 9 weeks post operation, but not at previous time points. There was no significant difference between cell/scaffold and autograft groups, which indicates that the efficacy of this cell/scaffold construct can compete with the gold standard of nerve repair.

**Table 1:** SFI values of the four experimental groups

Time (week)	Autograft	Cell/Scaffold	Scaffold	Silicone conduit
0	-95.3 ± 4.4	-105 ± 4.3	-100.7 ± 8.9	-94.7 ± 7.5
2	-81.1 ± 3.4	-86.4 ± 6.3	-88.9 ± 8.5	-90.4 ± 8.2
4	-74.3 ± 8.2	-69.5 ± 5.4	-84.2 ± 7.5	-82.7 ± 5.7
7	-59.7 ± 3.3*	-65.9 ± 4.1	-73 ± 6.1	-79.8 ± 6
9	-54.6 ± 6.1*	-61.9 ± 5.6*	-74.4 ± 4.7	-76.1 ± 8.9

Numbers are average ± SD. Footprints were taken from 4 to 6 rats per group. Rats showing heel ulcers were excluded from the test. “*” implies significant difference with scaffold only and silicone group

### Histological and morphometric studies

**Fig. 3:**
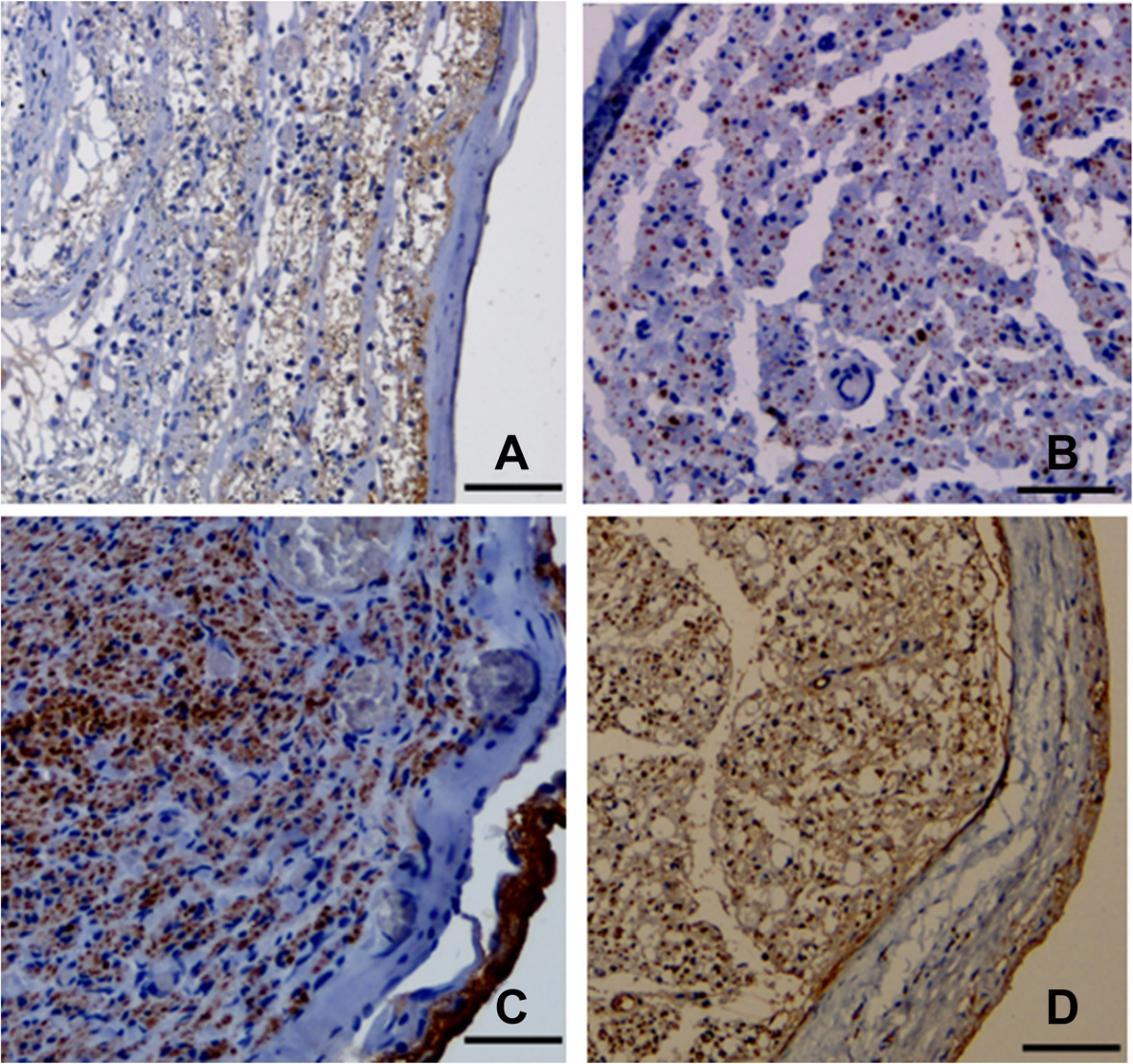
Light micrographs of NF immunostained sections of the grafts. The transverse sections were obtained from the midpoint of the nerve grafts. **a**: Silicone group, **b**: Scaffold group, **c**: Cell/scaffold group, **d**: Autograft group. Scale bars = 50 μm

The transverse sections at the middle portions of tissue engineered nerve grafts, silicone tubes and autografts were analyzed by NF and S100 immunostaining. NF expression is well correlated with axonal regeneration and consequently restoration of the nerve tissue. Although NF immunopositive nerve fibers were observable in all groups, but there was little nerve regeneration in the scaffold and silicone tube controls (Fig. 3a–d). In contrast noticeable expression of NF was observed in cell/scaffold and autograft groups. The area of regenerated nerve fibers in the control and scaffold group was considerably less than that in cell/scaffold and autograft groups. However, among the latter two groups, the amount of NF expression was more visibly pronounced in autograft group.

**Fig. 4:**
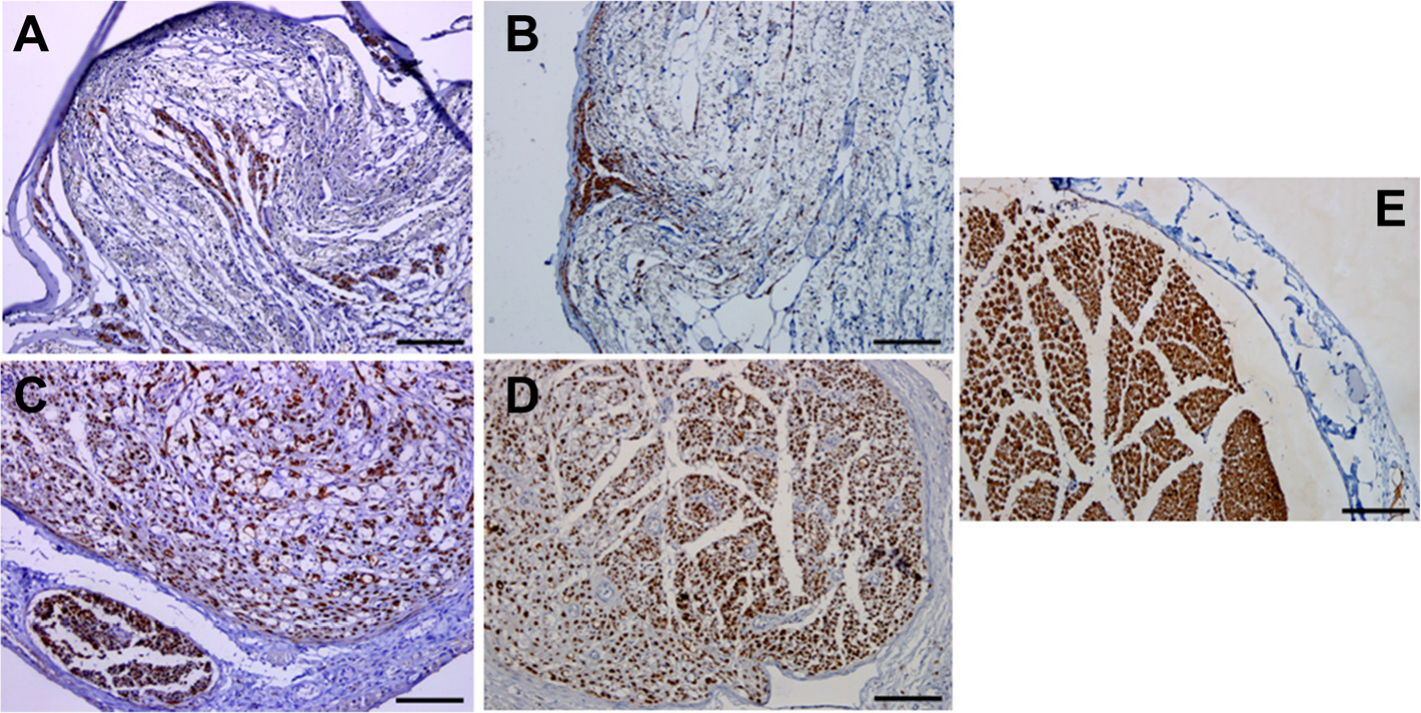
Light micrographs of S100 immunostained sections of the grafts. The cross sections were taken from the midpoint of the nerve conduit. **a**: Silicone group, **b**: Scaffold group, **c**: Cell/scaffold group, **d**: Autograft group and **e**: Normal sciatic nerve specimen; Scale bars = 50 μm

Immunochemistry with anti-S100 antibody indicates the regeneration of myelinated nerve fibers. S100 immunostained transverse sections were obtained from the middle portions of the grafts (Fig. 4a–e). In scaffold and silicone control groups, small and disperse areas of regenerated myelinated nerve fibers were visible. In contrast there was an even distribution of S100 immunostained area in autograft and cell/scaffold groups. Nevertheless, the organized structure of S100, as is shown in Fig. 4e in normal nerve tissues, is not observed in either autograft or cell/scaffold groups. Collectively, NF and S100 Immunochemical results indicate that OEC implantation promoted better nerve repair as evidenced by more axonal regeneration and myelination in the group receiving cell transplantation versus that with scaffold grafts with no cells included.

**Fig. 5:**
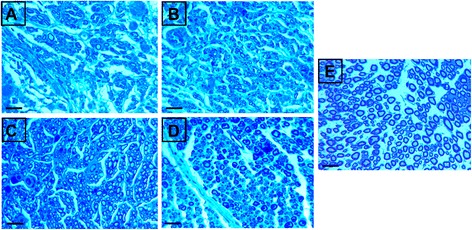
Light microscopy images of TB staining of transverse semithin sections from the distal part of the regenerated nerve stump. **a**: Silicone group, **b**: Scaffold group, **c**: Cell/scaffold group, **d**: Autograft group and **e**: Normal sciatic nerve as control; scale bars = 20 μm

**Fig. 6:**
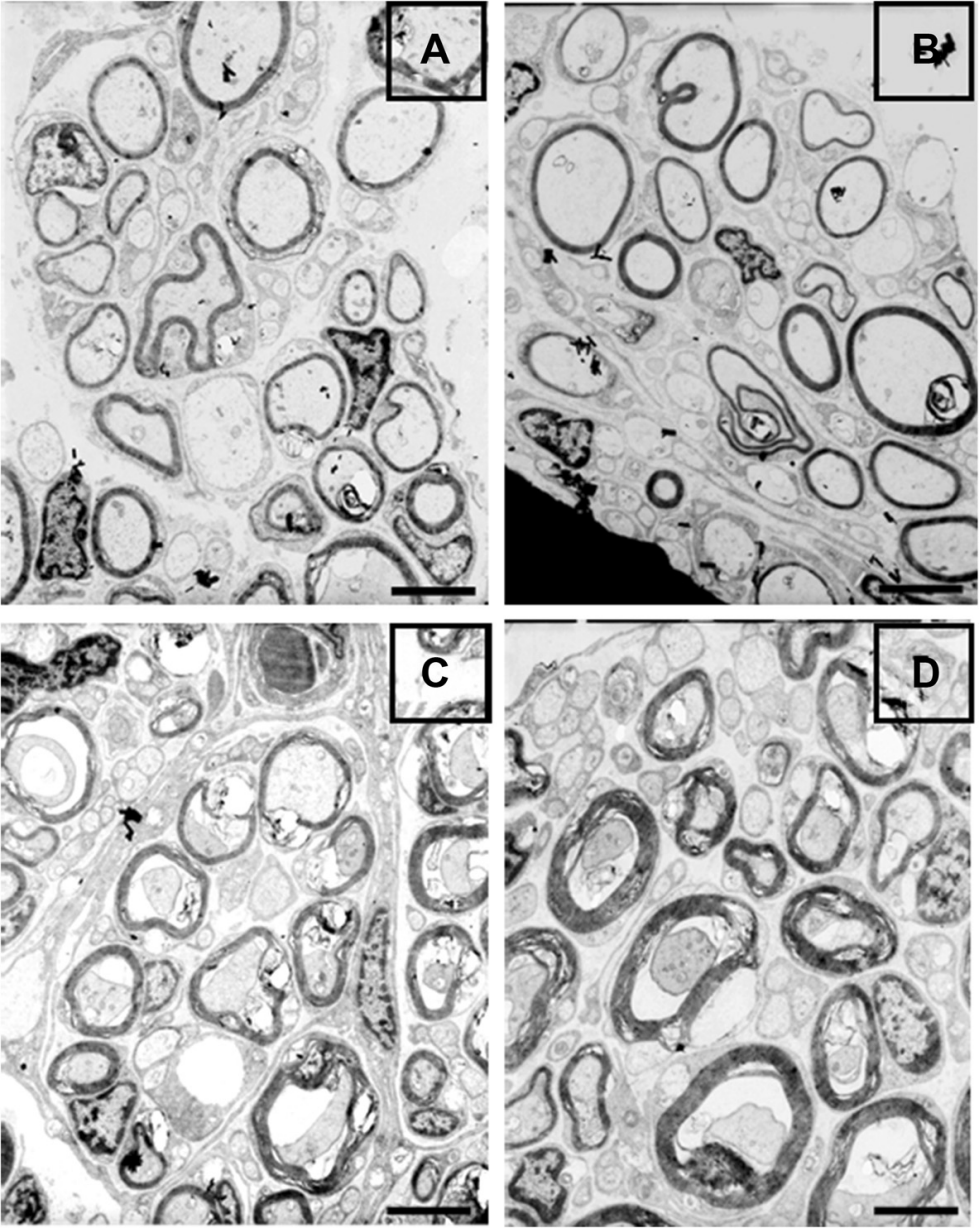
Transmission electron microscopy of ultra-thin sections of the regenerated nerves. The transverse sections were taken from the midpoint of the nerve conduit. **a**: Silicone group, **b**: Scaffold group, **c**: Cell/scaffold group, **d**: Autograft group; scale bars = 5 μm

Histological analysis of nerve regeneration on TB stained transverse semi-thin sections at the distal portion of the grafts or scaffolds revealed myelination of nerve fibers in all of the groups with differing degrees. The presence of myelinated nerve fibers shows that the regenerating axons have grown all the way through the graft and reached the distal nerve stump. In this study a few myelinated nerve fibers were visible in silicone conduit and scaffold groups (Fig. 5a, b). Myelinated nerve fibers are seen in higher densities in autograft and cell/scaffold groups, though immature myelination is still obvious in some parts (Fig. 5c, d). These observations reveal that inclusion of OEC can augment outgrowth of injured nerve across the nerve gap. Normal sciatic specimen shows uniform and thick myelin sheath with large nerve diameter (Fig. 5e). Transmission electron microscopy (TEM) provided further evidence for these observations. TEM micrographs of transverse ultrathin sections at the middle portions of the grafts revealed a similar trend in myelination of nerve fibers as that in distal parts of the nerve stumps (Fig. 6a–d). Similar to TB stained images, the myelinated axons in autograft group were surrounded with thicker and more electron dense myelin sheaths relative to other groups. The quantitative analysis of transverse sections further confirmed these findings.

Analysis of morphometric data from TB stained transverse sections At 9 weeks postoperatively, revealed significant differences in nerve fiber diameter, myelin sheath thickness and axonal diameter in autograph and cell/scaffold groups compared with silicone and scaffold groups (Table 2). Among the former two groups the diameter of the nerve fiber and the thickness of the myelin sheath were higher in autograft group, indicating that nerve fibers could better regenerate along the gap through the autograft than in polymer conduits. However, there was no statistical difference in axonal diameter between these groups. Also, in terms of morphology, the nerve fibers were surrounded by more uniform myelin sheaths in cell/scaffold group.

**Table 2:** Morphometric results of the regenerated nerve tissue after 9 weeks of implantation of nerve grafts

	Normal nerve	Autograft	Cell/Scaffold	Scaffold	Silicone
**Mean diameter of myelinated nerve fiber (μm)**	6.65 ± 0.63*	4.32 ± 0.45*	3.65 ± 0.31*	2.71 ± 0.27	2.56 ± 0.31
**Mean axoanl diameter (μm)**	4.02 ± 0.36*	2.83 ± 0.34*	2.54 ± 0.24*	1.7 ± 0.17	1.39 ± 0.12
**Mean thickness of myelin sheath (μm)**	1.32 ± 0.11*	0.78 ± 0.11*	0.58 ± 0.08*	0.45 ± 0.09	0.49 ± 0.06

* indicates significantly (*p* < 0.05) different with scaffold and silicone groups. Numbers are mean ± SD

### Cell survival

**Fig. 7:**
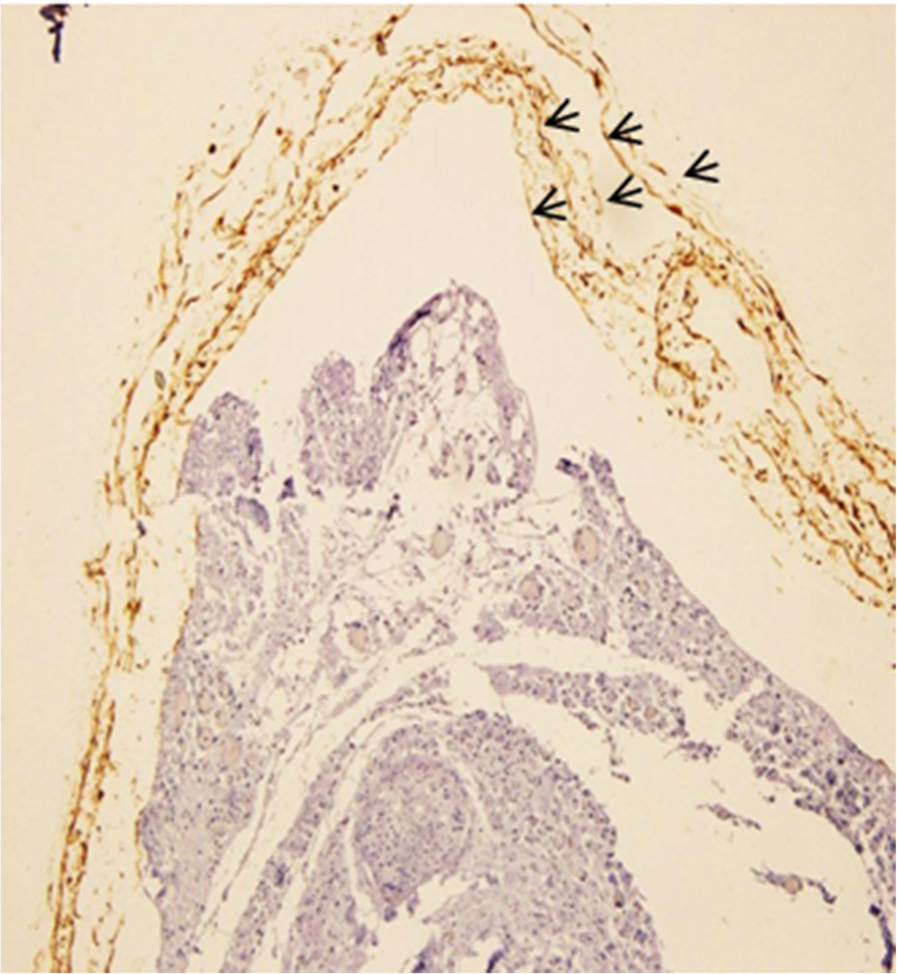
OEC survival after 9 weeks post transplantation. The presence of the cells is obvious in the peripheral areas of the nerve by brown color; arrows point to the cells located in adjacent layers of the conduit. Magnification 100x

A potential application of cells in nerve injury repairs is to promote axonal regeneration through their secretary neurotrophic factors. We observed OEC survival up to 9 weeks in rats after implantation of a cell-containing polymer scaffold in a complete transected sciatic nerve. The presence of cells in between the rolled electrospun sheets is obvious in Fig. 7.

## Discussion

The function of tissue engineered nerve graft is to encourage and guide regrowth between the ends of the severed axons. However, nerve regeneration is a complex phenomenon and requires a combination of different modalities such as surface architecture, contact guidance, appropriate physical and electrical properties and biological cues.

To assess the neurological performance of SWCNT doped PLLA nanofibrous conduit as a substrate for axon guidance and a platform for cell delivery, as well as the neuro-regenerative capability of OEC, 8 mm defects in sciatic nerves were bridged with the presented tissue engineered nerve conduits, with or without pre-seeded OEC.

Several synthetic biomaterials have been shown to enhance nerve regeneration. Among these poly (α-hydroxy esters) have been the early choices to make NGC. After implantation in sciatic nerve lesion, such conduits have resulted in an improved axonal regeneration relative to silicone rubber tubes [32]. Different strategies have been pursued to further tune their physical properties including inherent conductivity, surface texture and fiber dimensions, in order to make them more compatible with nerve regeneration purposes [33–35]. For example, enhanced conduction rates and peripheral nerve regeneration has been achieved through electrical poling of PLGA conduits [36]. Meanwhile the enhancing effect of electrical stimulation on neurite outgrowth and axon regeneration via using electrically conductive biomaterials has been documented [37, 25]. These studies highlight the facilitatory effect of electrical stimulation on nerve regeneration. It is shown that combining electrical stimulation and nanoscale features in biomaterials can elicit superior neural regenerative capacity [38]. However, few studies have addressed the combination of nanomaterials and such known electrical cues. In this study we exploited the biocompatibility and nanoscale features of electrospun PLLA fibers along with the inherent conductivity of CNT to fabricate conductive nanofeatured nerve conduits.

Cell incorporation into the nerve conduit is appreciated as an alternative to the exogenous application of growth factors. In this regard we took the advantage of thin electrospun sheets to be rolled and packed into a cylindrical shape in order to make a multi-shell hollow conduit capable to deliver repair promoting cells to the site of injury while having aligned micro-structured surface to direct newly sprouted axons to the distal stump of the truncated nerve via topographical cues. Since this scaffold was destined to serve as a NGC and a cell delivery platform, it was necessary to test its biocompatibility with the desired cell type. As such, the biological characteristics and proliferation capability of rat OEC were assessed before implementing *in vivo* studies (unpublished observation). The metabolic activity of the cells was slightly better on the composite scaffolds when compared to tissue culture polystyrene (TCP) surfaces. We observed that the plasma treated electrospun SWCNT/PLLA scaffolds were as suitable of a substrate for OEC as the widely used poly-lysine coated TCP plates. The preliminary studies to ensure the cytocompatibility of CNT/PLLA nanofibers showed no inhibitory effects on OEC survival and proliferation and proved the biocompatible nature of the composite scaffold that can be used safely as a nerve conduit material (unpublished observation). The OEC seeded on the aligned scaffold clearly adopt the strict longitudinal orientation of the substratum.

The continuous improvement in SFI implies that reinnervation of the muscles is establishing with time. Although the best average SFI score was observed in the group with autograft treatment, there was no significant difference between the autograft and cell/scaffold groups. In fact the high regeneration potential in autografts is likely due to the presence of neurotrophic factors in conjunction with aligned conduits [39]. Functional restoration after sciatic nerve injury was 17 % better in the combined cell/scaffold transplanted group than scaffold group. The SFI data revealed that after nine weeks post transplantation, functional restoration in cell/scaffold group reached that in autograft group. We observed significant improvement in SFI in the cell/scaffold group compared with scaffold alone and silicone groups after nine weeks, which indicates more successful motor function in the group that received OEC transplantation, underscoring the crucial roles of OEC. Similarly, in another study using neural stem cells as the supporting cells and chitosan-gold nanocomposites as the conduit material, the functional recovery was significantly greater in cell-seeded conduits than the group with no cells outlining the critical role of glial support cells in axonal regeneration [40].

Immunoreactivity to NF and S100 was observed with differing degrees in the cross sections obtained from regenerated nerve segments. In cell/scaffold and autograph groups S100 showed a dispersed expression across the nerve grafts, while in scaffold and silicone groups it was faintly positive and mostly had a local expression and these results resemble those for NF expression. Staining with anti-NF and S100 indicated that fibers regenerated through the scaffold conduit lumen were markedly less stained than in cell/scaffold group. A poor regenerative outcome was obvious in silicone and scaffold alone groups. Morphometric results indicated that the cell/scaffold group had more even but thinner myelin sheaths as compared to the autograft group.

Having a high aspect ratio electrospun nanofibers, especially in the form of a multi-shell layered conduit, can provide more available surface area for the cells to grow as compared to simple hollow film conduits; this translates into transplanting greater number of cells into the defect site. The long-term survival of implanted OEC was maintained within the nerve conduit. Enhanced functional and histological restoration in the group receiving OEC suggests that neurotrophic or induction effects associated with these cell types along with their synergistic interaction with Schwann cells in axon myelination might have led to this improved outcome [41]. We did not observe any difference regarding functional or anatomical restoration between the scaffold conduits and silicone groups, both showing inferior outcome when compared to cell/scaffold and autograft groups. These results are in line with previous studies reporting that PLGA nerve conduits, alone or with extra cellular matrix (ECM), cannot boost repair of rat sciatic nerve injuries [41], or another one showing that absence of aligned neuronal supporting cells within the conduit can lead to early degeneration of elongated axons [40, 42]. Thus the observed neural regeneration in cell/scaffold group can be mostly assigned to the presence of OEC. The implanted OEC serve to protect and support regenerating tissue. These cells have a high capacity in producing trophic factors while not expressing growth inhibitory proteoglycans [43, 44]. Meanwhile the composite multi-shell conduit can not only provide ample surface to transplant higher number of OEC, but assist the maintenance of locally secreted nerve growth factors from OEC which can promote glial migration from the nerve stump into the graft and also dictates both the direction of migration of endogenous cells and the newly sprouted axons through provision of contact guidance cues of aligned fibers [44]. OEC can not only guide the new forming axons across a route but may also help Schwann cells to infiltrate the graft [14]. Although OEC can myelinate axons [19], but the myelinating role of Schwann cells is likely more essential in this aspect of regeneration [41]. We rarely detected gfp OEC in the mid parts of cross sections of the grafts, however there was a marked expression of S100 in cell/scaffold group relative to cell free controls. Probably OEC contributed to this outcome by phagocytic removal of neuronal debris [45], eliciting axonal ingrowth through the scar tissue usually formed at the interface of nerve and graft and boosting their myelination by either OEC themselves or attracting SC towards the graft via their neurotrophic effects [17]. The increased S100 expression in cell/scaffold group is likely due to the synergistic cooperation between the two glial cell types in myelinating axons and promoting peripheral nerve repair [41].

## Conclusion

In this study we attempted to enhance the efficacy of tissue engineered nerve grafts by culturing OEC within the CNT/PLLA conduit walls to make bioactive artificial nerve grafts. After confirming the biocompatibility of the scaffold as a platform for cell delivery, we developed a novel tissue engineered nerve graft by introducing OEC on the walls of a CNT/PLLA nanofibrous multi-shell conduit and used it to bridge an eight mm gap in rat sciatic nerve. Our main finding in this study was that an OEC-seeded nerve conduit transplanted to the transected rat sciatic nerve could improve anatomical and functional recovery better than a nerve conduit without any grafted cells and with an efficacy close to that of nerve autografts. These findings support the fact that a combination of cell and biomaterial would enhance repair of sciatic nerve better than biomaterial alone and emphasizes the promise of OEC for transplantation to neural lesions. The novelty of this work lies in the application of SWCNT/PLLA nanofibrous conduits for peripheral nerve regeneration alone or in combination with known stimulatory cues of OEC. Taken all these results into account, we can conclude that transplantation of OEC in a conductive nanostructured conduit can efficiently improve functional and anatomical repair of severed sciatic nerve with potential application in development of tissue engineered nerve grafts for peripheral nerve repair.

## Abbreviations

(OEC): Olfactory ensheathing cells
(SWCNT): Single-walled carbon nanotube
(PLLA): Poly (_L_-lactic acid)
(SFI): Sciatic functional index
(NGC): Nerve guidance conduits
(PLGA): Poly (_L_-lactidecoglycolide)
(SCI): Spinal cord injuries
(SC): Schwann cells
(PNI): Peripheral nerve injuries
(DMF): Dimethylformamide
(SEM): Scanning electron microscopy
(PBS): Phosphate buffered saline
(TB): Toluidine blue
(TEM): Transmission electron microscopy
(TCP): Tissue culture polystyrene
